# Supplementary material for: Incidence of Hereditary Gastric Cancer May Be Much Higher than Reported
Source: Cancers (Basel). 2022 Dec 12;14(24):6125. doi: 10.3390/cancers14246125 (PMC9776697; doi:10.3390/cancers14246125)
Supplement: Supplementary file 1 [file cancers-14-06125-s001.zip › Supplementary files/Supplementary Table 2. Genes involved in the replication or repair processes (GRepRep)..pdf]

RepRep GENES

ALKBH2	CHEK1	FANCB	LIG4	OGG1	PRIM1	RFC1	TDP1	XRCC5
ALKBH3	CHEK2	FANCC	MAD2L2	PALB2	PRIM2	RFC2	TDP2	XRCC6
APE1	CLK2	FANCD2	MATR3	PARP1	PRIMPOL	RFC3	TOP1	
APEX1	CLSPN	FANCE	MBD4	PARP2	PRKDC	RFC4	TOP2A	
APEX2	CRY1	FANCF	MCM9	PARP3	PRPF19	RFC5	TOP2B	
APITD1	CUL4A	FANCG	MDC1	PARP4	PTTG1	RIF1	TOP3A	
APLF	CUL4B	FANCI	MGMT	PCNA	RAD1	RMI1	TOP3B	
APTX	DCLRE1A	FANCL	MLH1	PER1	RAD17	RMI2	TOPBP1	
ASF1A	DCLRE1B	FANCM	MLH3	PMS1	RAD18	RNF168	TP53	
ATM	DCLRE1C	FEN1	MMS19	PMS2	RAD21	RNF4	TP53BP1	
ATR	DDB1	FPG	MNAT1	PMS2L3	RAD23A	RNF8	TREX1	
ATRIP	DDB2	GADD45A	MPG	PNKP	RAD23B	RPA1	TREX2	
ATXN3	DMC1	GEN1	MRE11A	POLA1	RAD50	RPA2	UBE2A	
BCCIP	DNTT	GTF2H1	MSH2	POLB	RAD51	RPA3	UBE2B	
BL1	DUT	GTF2H2	MSH3	POLD1	RAD51B	RPA4	UBE2N	
BLM	EME1	GTF2H3	MSH4	POLD2	RAD51C	RRM2B	UBE2T	
BRCA1	EME2	GTF2H4	MSH5	POLD3	RAD51D	SETMAR	UBE2V1	
BRCA2	ENDOV	GTF2H5	MSH6	POLD4	RAD52	SFPQ	UBE2V2	
BRIP1	ERCC1	H1	MTH1	POLE	RAD54B	SHFM1	UNG	
BTBD12	ERCC2	H2AFX	MUS81	POLE2	RAD54L	SHPRH	USP1	
C17orf70	ERCC3	HELQ	MUTYH	POLE3	RAD9A	SLX1A	UVSSA	
C19orf40	ERCC4	HFM1	MYH	POLE4	RBBP8	SLX1B	WDR48	
C1orf86	ERCC5	HLTF	NBN	POLG	RBM14	SMC1A	WRN	
C7orf11	ERCC6	HMGB1	NEIL1	POLH	RBX1	SMC3	XAB2	
CCNH	ERCC6L	HMGB2	NEIL2	POLI	RDM1	SMC5	XPA	
CDK7	ERCC8	HTATIP2	NEIL3	POLK	RECQL	SMUG1	XPC	
CEBPG	ESCO2	HUS1	NHEJ1	POLL	RECQL4	SPO11	XRCC1	
CETN2	EXO1	KAT5	NTHL1	POLM	RECQL5	SPRTN	XRCC2	
CHAF1A	FAN1	LIG1	NUDT1	POLN	REV1L	SYCP3	XRCC3	
CHAF1B	FANCA	LIG3	OBFC2B	POLQ	REV3L	TDG	XRCC4	

Supplementary Table 2. Genes involved in the replication or repair processes (GRepRep).
