# Supplementary material for: Incidence of Hereditary Gastric Cancer May Be Much Higher than Reported
Source: Cancers (Basel). 2022 Dec 12;14(24):6125. doi: 10.3390/cancers14246125 (PMC9776697; doi:10.3390/cancers14246125)
Supplement: Supplementary file 1 [file cancers-14-06125-s001.zip › Supplementary files/Supplementary Table 1. Clinical and pathological data of the patients..pdf]

Sample	Region	Gender	Age	H.type	Stage	PCR.urease	CAGA	EBV	GMB	Age2	EBV_Urease	EBV_CAGA
CA130	cardia	male	36	intestinal	IV	positive	positive	negative	63	30<Age<40	positive	positive
CA133	body	male	31	diffuse	IV	positive	positive	negative	104	30<Age<40	positive	positive
CA134	cardia	male	38	diffuse	IV	positive	positive	negative	84	30<Age<40	positive	positive
CA14	NA	male	36	diffuse	III	NA	NA	NA	55	30<Age<40	NA	NA
CA142	antrum	male	28	diffuse	IB	positive	positive	negative	90	30-	positive	positive
CA149	cardia	male	35	intestinal	IIIB	positive	negative	negativo	69	30<Age<40	positive	negative
CA151	body	male	34	diffuse	IIIA	positive	positive	negative	42	30<Age<40	positive	positive
CA154	fundus	male	42	intestinal	IV	negative	negative	negativo	82	40+	negative	negative
CA173	body	female	45	intestinal	IV	positive	positive	positive	79	40+	positive	positive
CA175	antrum	male	47	intestinal	IIIB	positive	positive	negative	82	40+	positive	positive
CA195	antrum	male	47	intestinal	IIIA	positive	positive	positive	63	40+	positive	positive
CA208	antrum	female	49	intestinal	IV	positive	positive	negative	179	40+	positive	positive
CA211	cardia	female	34	diffuse	IV	positive	negative	negativo	52	30<Age<40	positive	negative
CA212	antrum	male	39	diffuse	IIA	positive	positive	positive	92	30<Age<40	positive	positive
CA213	body	female	39	intestinal	IV	positive	positive	negative	68	30<Age<40	positive	positive
CA218	antrum	female	48	diffuse	IV	negative	negative	negativo	135	40+	negative	negative
CA224	antrum	male	46	diffuse	IB	positive	positive	negative	75	40+	positive	positive
CA232	cardia	male	35	intestinal	IIA	positive	negative	negativo	97	30<Age<40	positive	negative
CA274	antrum	male	42	diffuse	IIIB	positive	positive	negative	44	40+	positive	positive
CA292	cardia	male	50	diffuse	IV	negative	negative	negativo	50	40+	negative	negative
CA302	antrum	female	33	diffuse	IV	positive	positive	negative	55	30<Age<40	positive	positive
CA304	body	male	34	diffuse	IIIA	positive	positive	negative	63	30<Age<40	positive	positive
CA362	cardia	female	47	diffuse	IIIA	positive	positive	negative	70	40+	positive	positive
CA394	cardia	male	47	diffuse	IV	negative	negative	negativo	69	40+	negative	negative
CA404	body	male	41	diffuse	IIIA	positive	positive	negative	68	40+	positive	positive
CA408	cardia	female	48	diffuse	IIIB	positive	positive	negative	87	40+	positive	positive
CA411	fundus	male	49	diffuse	IV	positive	positive	negative	38	40+	positive	positive
CA418	cardia	male	34	diffuse	IIIA	positive	negative	negativo	68	30<Age<40	positive	negative
CA419	body	male	46	diffuse	IIIA	negative	negative	negativo	53	40+	negative	negative
CA425	fundus	female	49	diffuse	IV	positive	negative	negativo	69	40+	positive	negative
CA427	cardia	male	48	diffuse	IV	positive	positive	negative	105	40+	positive	positive
CA429	antrum	male	35	diffuse	IIIA	positive	positive	negative	96	30<Age<40	positive	positive
CA430	antrum	male	46	diffuse	IIIA	positive	positive	positive	50	40+	positive	positive
CA439	antrum	female	27	diffuse	IIA	negative	negative	negativo	103	30-	negative	negative
CA440	antrum	female	28	diffuse	IV	negative	negative	negativo	88	30-	negative	negative
CA441	antrum	male	28	diffuse	IA	negative	negative	negativo	53	30-	negative	negative
CA442	antrum	female	29	diffuse	IV	negative	negative	negativo	99	30-	negative	negative
CA443	antrum	female	29	diffuse	IIIC	negative	negative	negativo	48	30-	negative	negative
CA444	antrum	female	30	diffuse	IIA	positive	positive	negative	104	30-	positive	positive
CA445	fundus	male	30	intestinal	IIIC	negative	negative	negativo	48	30-	negative	negative
CA446	antrum	male	30	intestinal	IIB	positive	positive	positive	90	30-	positive	positive
CA447	antrum	female	31	diffuse	IV	positive	positive	negative	94	30<Age<40	positive	positive
CA448	antrum	female	31	diffuse	IV	positive	negative	negativo	32	30<Age<40	positive	negative
CA449	antrum	female	32	diffuse	IIA	negative	negative	negativo	92	30<Age<40	negative	negative
CA450	antrum	male	32	intestinal	IV	negative	negative	negativo	91	30<Age<40	negative	negative
CA451	fundus	female	32	diffuse	IIA	positive	positive	negative	68	30<Age<40	positive	positive
CA452	antrum	male	33	intestinal	IV	positive	positive	negative	89	30<Age<40	positive	positive
CA455	fundus	female	34	diffuse	IV	positive	positive	negative	48	30<Age<40	positive	positive

CA456	antrum	female	35	diffuse	IV	positive	positive	negative	87	30<Age<40	positive	positive
CA457	antrum	female	36	diffuse	IIA	negative	negative	negativo	42	30<Age<40	negative	negative
CA458	fundus	male	37	intestinal	IV	negative	negative	negativo	103	30<Age<40	negative	negative
CA459	fundus	male	37	intestinal	IV	negative	negative	positive	48	30<Age<40	positive	positive
CA460	cardia	female	38	diffuse	IIA	positive	positive	negative	68	30<Age<40	positive	positive
CA462	antrum	female	39	intestinal	IIIA	positive	negative	negativo	94	30<Age<40	positive	negative
CA463	cardia	male	40	intestinal	IIIC	positive	positive	negative	68	40+	positive	positive
CA464	antrum	female	40	intestinal	IIA	negative	negative	negativo	85	40+	negative	negative
CA465	antrum	male	40	intestinal	IV	positive	positive	negative	16	40+	positive	positive
CA466	antrum	female	41	intestinal	IV	negative	negative	positive	76	40+	positive	positive
CA467	fundus	female	41	diffuse	IIIB	negative	negative	negativo	82	40+	negative	negative
CA468	antrum	female	42	diffuse	IV	positive	positive	negative	84	40+	positive	positive
CA469	fundus	female	42	diffuse	IV	positive	positive	negative	59	40+	positive	positive
CA470	cardia	male	43	intestinal	IV	positive	negative	negative	81	40+	positive	negative
CA471	fundus	female	43	intestinal	IIB	negative	negative	negativo	82	40+	negative	negative
CA472	fundus	female	44	intestinal	IIIC	negative	negative	positive	117	40+	positive	positive
CA474	cardia	male	44	intestinal	IIB	positive	positive	negative	59	40+	positive	positive
CA475	cardia	male	45	intestinal	IV	positive	negative	positive	61	40+	positive	positive
CA477	antrum	female	45	intestinal	IV	negative	negative	negativo	62	40+	negative	negative
CA479	antrum	female	46	diffuse	IIA	positive	negative	negativo	66	40+	positive	negative
CA480	fundus	female	46	intestinal	IIA	positive	positive	negative	90	40+	positive	positive
CA481	fundus	female	46	intestinal	IIA	positive	positive	negative	109	40+	positive	positive
CA482	cardia	female	47	intestinal	IIA	positive	positive	negative	96	40+	positive	positive
CA483	fundus	female	47	intestinal	IV	negative	negative	negativo	74	40+	negative	negative
CA485	cardia	female	49	intestinal	IV	positive	negative	negativo	91	40+	positive	negative
CA487	antrum	female	49	intestinal	IV	positive	negative	negativo	79	40+	positive	negative
CA489	cardia	female	32	diffuse	IIIA	negative	negative	negativo	93	30<Age<40	negative	negative
CA490	fundus	female	33	diffuse	IIB	positive	negative	negativo	48	30<Age<40	positive	negative
CA496	antrum	female	27	diffuse	IB	positive	positive	negative	66	30-	positive	positive
CA523	fundo	female	42	diffuse	IIIA	negative	negative	negativo	52	40+	negative	negative
CA524	antrum	male	41	intestinal	IA	positive	positive	negative	129	40+	positive	positive
CA687	Cardia	male	37	diffuse	IV	NA	NA	NA	25	30<Age<40	NA	NA
CA688	Cardia	male	35	diffuse	IV	NA	NA	NA	7	30<Age<40	NA	NA
CA691	fundus	female	34	diffuse	IV	NA	NA	NA	69	30<Age<40	NA	NA
CA693	Cardia	male	38	diffuse	II	NA	NA	NA	35	30<Age<40	NA	NA
CA695	antrum	female	37	diffuse	II	NA	NA	NA	15	30<Age<40	NA	NA
CA706	Cardia	male	31	diffuse	II	NA	NA	NA	7	30<Age<40	NA	NA
CA711	Cardia	male	33	diffuse	IIA	NA	NA	NA	0	30<Age<40	NA	NA
CA717	body	female	29	diffuse	IV	NA	NA	NA	1	30-	NA	NA
CA736	body	male	38	diffuse	IV	NA	NA	NA	35	30<Age<40	NA	NA
CA741	antrum	male	35	diffuse	IB	NA	NA	NA	54	30<Age<40	NA	NA
CA742	antrum	male	34	diffuse	IV	NA	NA	NA	54	30<Age<40	NA	NA
CA743	antrum	male	33	diffuse	IB	NA	NA	NA	55	30<Age<40	NA	NA
CA867	body	female	20	diffuse	III	NA	NA	NA	23	30-	NA	NA
CA940	body	female	25	diffuse	IA	NA	NA	NA	79	30-	NA	NA
CA946	body	male	38	diffuse	IB	NA	NA	NA	70	30<Age<40	NA	NA
CA950	body	female	35	diffuse	IA	NA	NA	NA	23	30<Age<40	NA	NA

Supplementary Table 1. Clinical and pathological data of the patients.
